# Use of Microwave Technology for Agro-Based Polymers: A Selective Review

**DOI:** 10.3390/polym18091103

**Published:** 2026-04-30

**Authors:** Huai N. Cheng, Atanu Biswas, Michael Appell, Heping Cao, Zhongqi He

**Affiliations:** 1 USDA Agricultural Research Service, Southern Regional Research Center, New Orleans, LA 70124, USA; heping.cao@usda.gov (H.C.); zhongqi.he@usda.gov (Z.H.); 2 USDA Agricultural Research Service, National Center for Agricultural Utilization Research, Peoria, IL 61604, USA; atanu.biswas@usda.gov (A.B.); michael.appell@gmail.com (M.A.)

**Keywords:** microwave, polymer reactions, extractions, biobased materials, agro-based polymers, green chemistry, sustainability

## Abstract

Microwave technology is being used increasingly in polymer processing, where significant time and energy savings have been demonstrated across many systems. In this work, we first provide an overview of microwave-assisted processes involving agro-based materials, with emphasis on microwave-assisted modification reactions and extractions. A more detailed review then highlights several examples from the authors’ laboratories. For example, microwave heating has been shown to greatly accelerate the synthesis of cellulosic derivatives from cellulose and the formation of a polyurethane from a carbohydrate and a diisocyanate, while still producing polymers comparable in structure to those obtained by conventional heating. Likewise, microwave treatment can speed up pericyclic reactions involving triglycerides and cardanol, leading to products with enhanced viscosity. In extraction applications, such as recovering phenolic compounds from common beans, microwave methods can sometimes yield higher extraction efficiencies. Beyond time and energy savings, the reduced processing duration also decreases workers’ exposure to chemicals and solvents, thereby improving safety and lowering chemical hazards. Thus, microwave treatment can be considered a “green”, energy-efficient tool for many polymer reactions and processes.

## 1. Introduction and Overview

Microwave-assisted heating has become a widely adopted tool in polymer chemistry over the past 30 years because it often compresses reaction times lasting several hours into tens of minutes and can enable reactions that are difficult under conventional conditions. Researchers have applied microwave energy across many polymer reactions—ring-opening polymerization, controlled/”living” radical methods, polycondensations, curing and post-polymer modifications—and several reviews and experimental studies document consistent, large reductions in reaction time and, in some cases, improved molecular-weight control or product properties when carefully optimized [1,2,3,4].

Microwave heating efficiency in chemical reactions is fundamentally governed by how effectively a material converts electromagnetic energy into heat, which is primarily determined by its dielectric properties—the dielectric constant (ε′), dielectric loss (ε″), and especially the loss tangent (tan δ). Materials with higher tan δ values couple more efficiently with microwave radiation, leading to faster heating [5,6]. This dielectric response depends strongly on molecular characteristics, including dipole moment and ionic conductivity, as well as the composition of the reaction medium, such as solvent polarity, presence of salts, and phase heterogeneity. In addition, microwave parameters—including frequency, electric field strength, and applied power—directly influence the rate of energy absorption, while temperature plays a critical role because dielectric properties themselves vary with temperature, sometimes leading to nonlinear heating behavior or thermal runaway [7,8].

Beyond intrinsic material properties, physical and reactor-related factors significantly influence heating efficiency. These include penetration depth, which determines how uniformly energy is distributed within the sample, and depends on dielectric properties and frequency. Sample size, geometry, and mixing conditions affect field distribution and heat transfer, while reactor design (e.g., single-mode vs. multimode cavities) governs the uniformity of microwave exposure [9,10]. The presence of microwave absorbers or susceptors (such as carbon-based materials) can enhance heating in otherwise poor absorbers. Finally, thermal properties (heat capacity and thermal conductivity) influence how absorbed energy translates into temperature rise and distribution, and reaction-specific factors—including catalysts, kinetics, and selective heating of components—determine how efficiently the generated heat drives the desired chemical transformation [9,10,11]. Together, these interconnected factors define the overall efficiency and effectiveness of microwave-assisted chemical processes.

In the literature, two microwave effects are most often cited: the thermal microwave effect (where microwaves are a more efficient way to heat the reaction mixture) and specific (or non-thermal) microwave effects (effects not attributable to measured bulk temperature differences or where unexplained enhancement in reactivity or selectivity is observed) [4,12,13]. There is an active literature debate about the specific (non-thermal) microwave effects: some groups report lowered apparent activation energies or changed kinetics under microwave fields, while others indicate that careful temperature measurement and matched thermal profiles explain most rate differences [13,14,15,16]. Interpreting kinetic advantages, therefore, requires rigorous control of temperature, stirring, and reactor geometry. One of the viewpoints [17,18,19] points out that for many samples, the microwave energy is transferred faster to a sample than the molecules can relax, which results in a non-equilibrium condition and high internal instantaneous heating (causing micro-scale hot-spots) that may enhance the reaction rate as well as the product yields; this may perhaps be a possible mechanism for the observed rate enhancements.

In practical terms, microwave-assisted reactions have some clear advantages over conventional conductive/convective heating, viz., often faster heating and shorter reaction times (thereby higher throughput and lower energy consumption), selective heating of reactants/solvents that can improve yields or material properties, and convenient solventless or solvent-reduced protocols that fit green-chemistry goals. Disadvantages and limitations include: scale-up and reproducibility challenges (microwave field distribution and cavity design matter, so lab successes can be hard to translate to large scale), limited penetration depth in highly viscous or low-dielectric materials, requirement for polar/absorbing species (thus, non-polar or non-absorbing materials needing special additives), the potential for local “hot-spots” or runaway if temperature is not monitored properly, equipment cost, and safety considerations for pressurized/closed systems [20,21,22,23].

Agro-based materials are gaining traction as replacements for petroleum-derived feedstocks because they offer sustainability, environmental friendliness, improved recyclability, and low toxicity [24,25,26,27,28]. However, their inherent physical properties often fall short of the requirements for many industrial polymer applications. As a result, chemical modification or physical processing is typically necessary to achieve suitable performance [29,30,31,32]. Unlike synthetic polymer chemistry, where microwave heating often promotes polymerization or curing, much of the work on biobased systems includes the addition of function groups or the use of specific reactions. Examples include esterification, etherification, grafting, hydrolysis, crosslinking, and hydrolysis of polysaccharides and proteins, as well as transesterification of fats and oils. These approaches have enabled the development of various products, e.g., bioplastics, adhesives, caulks, packaging films, textiles, encapsulants, thickeners, surfactants, and food-related materials [24,25,26,33,34].

Microwave-assisted chemistry has become an important tool for modifying agro-based polymeric materials because of its ability to accelerate reactions and improve efficiency. For ease of reference, examples of several classes of agro-based materials that have involved microwave-assisted modifications are summarized in Table 1. A popular class of materials for these modification reactions includes the polysaccharides, and several review articles have appeared [35,36,37,38,39]. Among them, starch has received a lot of attention. In fact, microwave has often been used on starch itself without chemical reactants in order to improve its properties [40,41,42,43]. As for chemical modifications [44,45], some of the starch derivatives made with the help of microwave include hydrolyzed starch [46], starch acetate [47,48,49,50,51], starch maleate [49] and malate [52], inorganic esters of starch [53,54], other starch esters [55], oxidized starch [56,57], and starch ethers, such as hydroxypropyl starch [58] and carboxymethyl starch [59].

Another well-studied area for microwave-assisted reactions involves cellulose. Cellulosic derivatives obtained with the assistance of microwave include oxidized cellulose [60,61,62], cellulose acetate [63], other cellulose esters [64], cellulose succinate [65], para-aminobenzoate [66], gallate [67], carbamate [68], and cellulose-urea adducts [69]. Cellulose ethers have also been made with the help of microwave, including methyl and ethyl cellulose [70] and carboxymethyl cellulose [71,72,73]. A few cellulosic derivatives involve the incorporation of poly(ethylenimine) [74,75,76]. Other derivatives have been obtained through graft reactions with 2-(dimethylamino)ethylmethacrylate [77], acrylamide [78], acrylic acid [79], and cyclodextrin [80].

Oils and fats represent another opportunity for microwave-assisted reactions [81]. A well-known reaction is trans-esterification in triglycerides [82,85] and biodiesel [83,84]. Another reaction entails ester formation to yield glycerol monoester [86] and medium-chain triglycerides [87]. Other useful reactions involve the conversion of olefins to epoxides [88,89] and the conversion of epoxides to polyol [90]. Soybean oil has been subjected to the ene reactions to form different ene reaction derivatives [91,92,93,94]. Moreover, triglycerides can be hydrolyzed via microwave to fatty acids [95].

Proteins comprise another major class of agro-based polymers, and microwave treatments have been used even without chemical reactants in order to improve their properties [99,100,101,102]. Some of the microwave-assisted reactions and related considerations have been reviewed [103,104,105]. An important reaction for food chemistry is the Maillard reaction, which can be accelerated with the help of microwave treatments [106,107,108]. Other microwave reaction products include protein-saccharide grafts [109], protein-ferulic acid adduct [110], and phosphorylated protein [111].

Table 1 gives more examples of agro-based materials that are amenable to microwave treatments. A promising opportunity is the reaction between a carbohydrate and a diisocyanate, which produces a biodegradable polyurethane [123,124,125,126,127,128]. Furthermore, cardanol is a byproduct from cashew nut processing [96,97], and it can be converted into ene reaction products through a microwave-assisted process [98]. Moreover, several references have reported on the microwave treatments of biomass and lignocellulosic residues [112,113,114,115,116,117,118], particularly pyrolysis products [116,119], and various modification reactions [70,72,120,121,122]. Another major application of microwave technology is the extraction of valuable components from agri-food materials [129,130,131,132,133,134,135,136,137].

Microwave processing of agro-based polymers offers the same advantages as the processing of synthetic polymers. Thus, microwave irradiation enables rapid heating of polar functional groups and heterogeneous reaction media, often converting multi-hour conventional reactions into processes lasting only minutes. In addition, the volumetric and selective nature of microwave heating leads to uniform temperature distribution, minimizing degradation and improving control of reaction selectivity. Nevertheless, challenges remain in applying microwaves to agro-based polymer modifications. The heterogeneous nature of many natural materials—often containing bound water, fibrous structure, and varying dielectric properties—can result in uneven heating or localized overheating, leading to degradation or inconsistent product quality. Scale-up beyond laboratory or pilot levels also remains difficult because of penetration-depth limitations and nonuniform electromagnetic fields [20,113,114,115]. Despite these drawbacks, the last 30 years of research have established microwave-assisted modification as a powerful approach to value-addition of agricultural byproducts and renewable polymers, offering significant energy savings, reduced processing time, and pathways to environmentally friendly material development.

In the following sections, a review of microwave-assisted modification reactions and extractions involving selected agro-based polymers is given to illustrate their utility, the range of different materials studied, and the increased reaction rates achieved, with a special emphasis on the work done in the authors’ laboratories. It is hoped that these examples will stimulate further application of microwave technology as a tool for green polymer chemistry processes and a valuable technique for agro-based polymer applications.

## 2. Microwave-Assisted Modification of Polysaccharides

As noted in the above section, microwave treatments have a notable impact on the derivatization of polysaccharides. Starch and cellulose are the polymers most often derivatized. Specific functional groups are usually attached to the polysaccharide structure in order to impart special properties [35,36,37,38,39]. A large number of these functional groups have been employed, as shown in Table 1.

**Figure 1:**
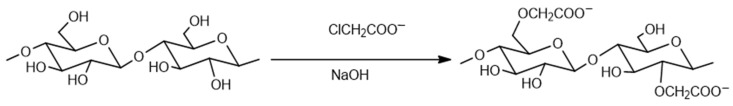
Conversion of cellulose to carboxymethyl cellulose (CMC).

In view of continuing interest in the utilization of agricultural byproducts, we have earlier converted microcrystalline cellulose (m-cellulose), cellulose fiber (f-cellulose), wheat straw, barley straw, and rice hull into carboxymethylcellulose (CMC), using the same procedure involving the formation of alkali cellulose in an alcohol and the addition of monochloroacetic acid [72] (Figure 1).

It was found that the three agro-based materials produced roughly the same degree of substitution as the two cellulose samples (Table 2, lines 1–5). We also used a microwave for the cellulose samples instead of conventional heat, enabling an improved synthesis of CMC to be conducted, with similar degrees of substitution (DS) as conventional heat (3 h) but much reduced reaction times (30 min) (Table 2, lines 6–7) [72]. In order to gain a better understanding of the microwave-assisted mechanism, the results for the microwave reaction at 85 °C are also shown, where a reaction time of only 3 min was needed to achieve similar DS values (Table 2, lines 8–9). This result indicates the critical role of temperature for this microwave-assisted reaction.

Alkyl celluloses (e.g., methyl cellulose and ethyl cellulose) are commercial products that are typically made in alcoholic alkali cellulose with dialkyl sulfate or alkyl iodide over the course of several hours. We have used an alternative procedure involving microwave heating, which reduced the reaction time. The synthesis of methyl cellulose and ethyl cellulose was confirmed by ^13^C NMR and FT-IR analyses [70].

## 3. Triglycerides and Related Materials

Triglycerides and related materials (such as cardanol) are amenable to the use of microwave technology. Quite a few reports have appeared on the modifications of these materials [81]. We are highlighting here some projects from our labs where polymers are produced from the reactions.

### 3.1. Ene Reaction Products from Cardanol

**Figure 2:**
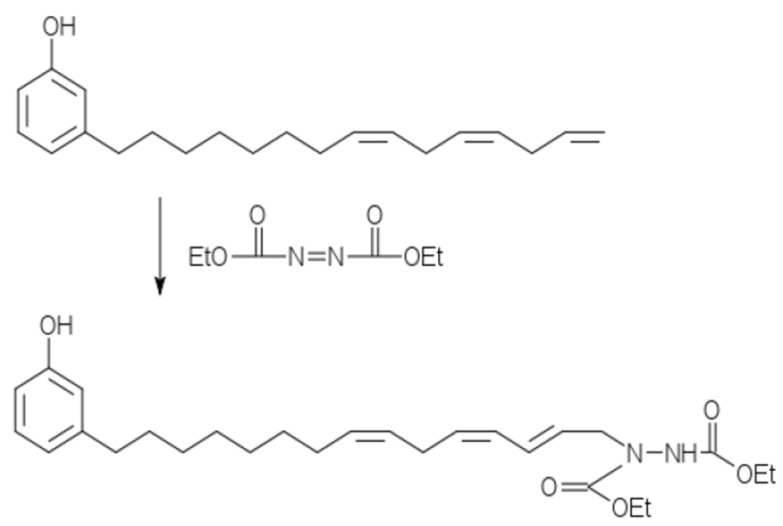
Schematic reaction of cardanol with DEAD. Cardanol contains the phenol structure and a hydrocarbon substituent containing 0, 1, 2, or 3 olefins. For illustration, only the triene substituent and only one possible reaction with DEAD are shown.

Cardanol, a product from cashew nut processing, contains three reactive sites, including phenolic OH, benzene ring, and olefins in the unsaturated side chain. Modifications of these reactive sites have been reviewed previously [96,97]. An interesting reaction involving cardanol is Alder’s ene reaction [138]. We reported earlier [98] the ene reaction between the olefins in cardanol and diethyl azodicarboxylate (DEAD) (Figure 2).

As shown in Table 3, the reaction took 6 h via conventional heating, but only 5 min with microwave. It is of interest that this cardanol-DEAD product increases in viscosity with increasing reaction time, probably due to the formation of Diels–Alder crosslinks [98], similar to the soybean oil-DEAD derivatives given in Section 3.2.

### 3.2. Ene Reaction Products from Triglycerides

Soybean oil provides another example of ene reactions involving DEAD [91]. For this reaction, DEAD and soybean oil were mixed in the absence of a catalyst and solvent. With the microwave, the reaction was completed in 5–15 min at 110 °C. With conventional heat, the reaction took longer at 60–110 °C. In both cases, the same soybean oil-DEAD adducts were obtained, and the products appeared to be very viscous, honey-colored oil [91].

It was also discovered that this soybean oil-DEAD ene reaction occurred gradually in 2–3 days at room temperature, and the mixture also increased its viscosity slowly over time (up to two weeks) due to the formation of a Diels–Alder reaction [92]. This self-curing system may be used, perhaps as a lubricant additive, thickener, adhesive, or cement application. A similar ene reaction of soybean oil with 4-phenyl-1,2,4-triazoline-3,5-dione (PTAD) was also reported [93]. Separately, Alarcon et al. [94] published a paper on the microwave-assisted ene reaction between triglyceride and maleic anhydride.

## 4. Proteins

In the literature, there is some interest in using microwave treatment for the protein itself without the use of chemical reagents in order to change its mechanical properties [99], antioxidant activity [100], and digestibility [100,101,102]. Many food manufacturers are now reducing their use of chemical protein modifications because consumers prefer clean-label products and because scaling up these processes can raise toxicity concerns. Even so, certain modification techniques remain useful, as they can speed up reactions and produce proteins with enhanced properties [103,104,105]. Some of the microwave-assisted reactions involving proteins have been shown in Table 1 and discussed in an earlier section.

## 5. Polymerization

**Figure 3:**
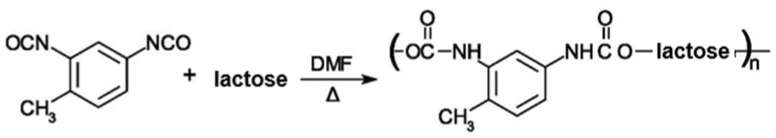
Reaction of lactose and toluene diisocyanate.

In addition to polymer reactions, microwave heating has been used effectively for polymerization. One area of our particular interest is the synthesis of polyurethanes from a carbohydrate and a diisocyanate. These include the polyurethanes made from cyclodextrin [123], starch [124], xylan [125], sucrose [126], lactose [127], and oligosaccharides [128]. The reaction of lactose and toluene diisocyanate (TDI) is shown in Figure 3.

Given in Table 4 are the lactose-TDI polymerization data at 145 °C for conventional heat and microwave [127]. It can be seen that microwave can achieve the same reaction in about 3 min, whereas conventional heat takes 20 min. The products are shown to have similar yields and chemical structures, using ^13^C NMR, TGA, and DSC. The product was a liquid, a viscous liquid, or a solid, depending on the TDI/lactose ratio. We also made semi-interpenetrating polymer networks with the lactose polyurethane near its gel point, embedding either poly(lactic acid) or poly(vinyl pyrrolidone) into the network [127].

## 6. Microwave-Assisted Extraction of Specific Natural Components

There is growing interest in extracting natural components—such as bioactives, nutraceuticals, flavors, and essential oils—from agro-based materials [139,140,141,142,143] because of their health, nutritional, cosmetic, and other beneficial properties. Among the various extraction methods available, microwave-assisted extraction [129,130,131,132,133] has emerged as a particularly effective and efficient approach for obtaining these valuable substances. Examples from the authors’ work include the extraction of phenolics from common beans [134,135] and the removal of non-starch oligosaccharides from ripe banana peels using natural deep eutectic solvents [136,137].

In an informative study [134], four temperatures (25, 50, 100, and 150 °C) and three solvents (water, 50% ethanol in water, and 100% ethanol) were used for the extraction of phenolics in eight common beans. The most effective extraction was achieved at a temperature of 150 °C using 50% ethanol. In particular, microwave-assisted extraction at 150 °C and 50% ethanol in water produced 2–3 times more phenolics than conventional heat extractions [134]. Furthermore, the extracted phenolic levels were higher in bean hull than in cotyledon, and higher in dark-colored beans versus light colored beans [134,144,145]. For illustration, the data for 100 °C extraction with water are shown in Table 5. The phenolic level for microwave treatment averaged 23.4 mg per g bean hull and 5.15 mg per g bean cotyledon (in gallic acid equivalents, GAE), but the corresponding averages for conventional heat treatment were 12.09 mg/g and 1.81 mg/g (Table 5). To some extent, similar results were obtained from antioxidant assays [135]. In view of the high phenolic content and the ease of extraction, the phenolic antioxidants from common beans may perhaps be used as potential natural additives in food items or in food packaging.

## 7. Comments

In the five examples discussed above, the use of microwave consistently reduced reaction times and lowered energy consumption compared with conventional heating. Although the benefits of microwave-assisted reactions are well established, debate persists regarding the existence of specific (non-thermal) microwave effects, as noted earlier in the Introduction. Across the case studies presented in Section 2, Section 3, Section 4, Section 5 and Section 6, a clear trend emerges: temperature plays a critical role in each process. For instance, in the conversion of cellulose to CMC, higher microwave temperatures led to shorter reaction times. Similarly, in the microwave-assisted extraction of phenolics from common beans, increased temperatures resulted in higher total phenolic yields. The influence of temperature was also evident in the ene reaction between soybean oil and DEAD, and it is well documented in the literature for polyurethane synthesis [146,147,148]. Given the critical role of temperature in governing reaction rates, these observations are not inconsistent with the viewpoint [17,18,19] that non-equilibrium conditions and high instantaneous local heating under microwave irradiation may contribute to the enhanced reactivity and the reduced reaction times. However, while this perspective is certainly plausible, additional comprehensive research is needed to fully validate it.

Currently, most reported microwave-assisted reactions have been carried out using bench-scale reactors. This naturally raises the question of whether such reactions can be feasibly scaled up. In fact, the potential market for industrial microwave reactors is substantial [149,150]. However, several technical challenges remain in designing and manufacturing large-scale microwave systems [149,151,152]. These include achieving uniform heating across large material beds, ensuring radiation safety and preventing runaway exothermic reactions, and the absence of universally accepted industrial protocols for microwave-assisted processes, which can complicate regulatory approval.

Despite these obstacles, notable progress has been made. For example, Industrial Microwave Systems addressed the limited penetration depth of microwaves—typically only a few centimeters in most liquids—by shifting from the standard household microwave frequency of 2450 MHz to 915 MHz. This lower frequency enables deeper penetration and supports much higher power levels in industrial applications [153]. In chemical manufacturing, continuous-flow systems offer another practical route to overcoming scale-up limitations. A prominent example is Japan’s Microwave Chemical Company [154], which operates large-scale industrial microwave production facilities. Several other companies are likewise engaged in scaling up microwave-assisted processes or supplying the necessary equipment [151,155,156,157]. A comparative overview of commercial microwave reactors designed for scale-up was published a few years ago [158].

## 8. Conclusions

Microwave technology is an effective tool for advancing sustainability and green polymer chemistry. By significantly accelerating reaction rates, microwave heating can reduce both energy consumption and processing time. Faster reactions also mean shorter handling periods, which can in turn lessen workers’ exposure to chemical reagents and improve overall laboratory safety. In our work, we have found microwave-assisted methods to be particularly valuable for polymer reactions and extractions of valuable natural components involving agro-based polymeric materials.

The specific examples highlighted in this review serve to illustrate these advantages. For instance, the synthesis of carboxymethyl cellulose (CMC) from agricultural byproducts can be completed much more rapidly under microwave irradiation—approximately 30 min, compared with 3 h using conventional heating. Similarly, cardanol reacts with DEAD to form ene and Diels–Alder adducts with enhanced viscosity. Under traditional heating at 70 °C, this transformation requires about 6 h, whereas microwave processing completes it in 5 min. Another example is the formation of polyurethanes from lactose and toluene diisocyanate at 145 °C, which takes 20 min with conventional heat but only 3 min under microwave conditions.

Beyond polymer reactions, microwave technology can also be a powerful tool for extraction. It is particularly useful for releasing targeted components from polymeric or biological matrices. In our work, we have successfully applied microwave-assisted extraction to isolate specific ingredients from food and agricultural materials—for example, extracting phenolics from common beans with improved efficiency and reduced processing time relative to conventional heat extraction.

Overall, microwave-assisted chemistry offers a versatile platform that supports greener, faster, and often more selective transformations in agro-based materials. When applied selectively, it can contribute significantly to sustainable practices in both research and industrial settings.

## Figures and Tables

**Table 1:** Microwave-assisted chemical modifications of selected agro-based polymers.

Polymer Types	Starting Materials	Modified Polymers	Refs.
Polysaccharides	Polysaccharides, general	Review of polysaccharide derivatives	[35,36,37,38,39]
Starch	Starch, general	Review—Microwave only, no chemical reactants	[40,41,42,43]
	Starch, general	Review—chemical derivatives	[44,45]
	Faba bean starch	Acid hydrolysate	[46]
	Corn Starch	Starch acetate	[47,48]
	Corn Starch	Starch acetate and maleate	[49]
	Potato starch	Starch acetate	[50]
	Wheat starch	Starch acetate	[51]
	Potato, sweet potato, pea starches	Starch malate	[52]
	Potato starch	Starch sulfate, borate, silicate, selenate, zincate	[53]
	Potato starch	Inorganic esters	[54]
	Corn starch	Branching, esterification	[55]
	Starch	Oxidation	[56]
	Talipot starch	Oxidation, esterification, crosslinking	[57]
	Corn starch	Hydroxypropyl ether	[58]
	Potato starch	Carboxymethyl ether	[59]
Cellulose	Bacterial cellulose	Periodate oxidation	[60]
	Cellulose hydrogel beads	TEMPO oxidation	[61]
	Microcrystalline cellulose	Oxidative degradation	[62]
	Cellulose from *Caragana korshinskii*	Cellulose acetate	[63]
	Waste cotton fabric	Cellulose esters	[64]
	Microcrystalline cellulose	Cellulose succinate	[65]
	Cellulose nanofiber	Cellulose para-aminobenzoate	[66]
	Cellulose amino derivative	Cellulose gallate	[67]
	Cotton linter, reed, bagasse, wood pulp	Cellulose carbamate	[68]
	Sugar cane bagasse	Urea derivative	[69]
	Microcrystalline cellulose	Methyl cellulose, ethyl cellulose	[70]
	Cotton stalk	CMC	[71]
	Agric residues	CMC, CMC acetate	[72]
	Cellulose from brewer’s spent grain	CMC	[73]
	Cellulose fiber	Poly(ethylenimine) modification	[74]
	Cellulose from ag waste	Poly(ethylenimine) modification	[75]
	Microcrystalline cellulose	Modified with succinate and poly(ethylenimine)	[76]
	Cellulose from sugarcane bagasse	2-(Dimethylamino)ethyl-methacrylate graft	[77]
	Microcrystalline cellulose	Acrylamide graft	[78]
	Carboxymethyl cellulose	Poly(acrylic acid) graft	[79]
	Rice husk cellulose	Cyclodextrin graft	[80]
Oils and Fats	Vegetable oils, general	Review—Modifications	[81]
	Triglycerides, general	Review—Triglyceride trans-esters	[82]
	Biodiesel	Biodiesel trans-esters	[83]
	Biodiesel	Biodiesel trans-esters	[84]
	Biodiesel and waste frying oil	Trans-esters	[85]
	Decanoic acid	Glycerol monoester	[86]
	Lauric acid	Medium-chain triglycerides	[87]
	Oils and fatty acids	Epoxides	[88,89]
	Epoxidized soybean oil	Soybean oil polyol	[90]
	Soybean oil	Soybean oil-DEAD derivative	[91,92]
	Soybean oil	Soybean oil PTAD derivative	[93]
	Soybean oil	Soybean oil maleic anhydride adduct	[94]
	Triglycerides	Free fatty acids	[95]
Cashew	Cardanol	Review of derivatives	[96,97]
	Cardanol	Cardanol-DEAD derivative	[98]
Proteins	Wheat protein	Microwave only, no chemical reactants	[99]
	Quinoa protein	Microwave only, no chemical reactants	[100]
	Spirulina platensis protein	Microwave only, no chemical reactants	[101]
	Pigeon pea flour	Microwave only, no chemical reactants	[102]
	Protein, general	Review, protein modifications	[103,104,105]
	Pea protein	Maillard products	[106]
	Rice protein	Maillard products	[107]
	Bovine serum albumin	Maillard products	[108]
	Soy protein	Protein-saccharide graft	[109]
	Soy protein	Protein-ferulic acid adduct	[110]
	Mung bean protein	Phosphorylation products	[111]
Biomass, Lignocellulose	Lignocellulose, general	Reviews—microwave treatments of biomass, lignocellulose	[112,113,114,115,116,117,118]
	lignocellulose	Pyrolysis products	[116,119]
	Natural fibers	Review—fiber modifications	[120]
	Jute fiber	Reaction with 1,2,4,5-benzene-tetracarboxylic anhydride (PMDA)	[121]
	Cotton gin trash	Reaction with maleic anhydride	[122]
	Agro byproducts	Various reactions	[70,72]
Carbohydrate polyurethanes	Cyclodextrin	Cyclodextrin-polyurethanes	[123]
	Starch	Starch-polyurethanes	[124]
	Xylan	Xylan-polyurethanes	[125]
	Sucrose	Sucrose-polyurethanes	[126]
	Lactose	Lactose-polyurethanes	[127]
	Oligosaccharides	Oligosaccharide-polyurethanes	[128]

**Table 2:** Results of carboxymethylation of cellulose and agro-based materials using conventional heat and microwave (adapted from ref. [72]).

Sample	Start Material	T(°C)	Time	Energy	DS
CX-3	m-cellulose	45	3 h	Conventional heat	0.8
CX-4	f-cellulose	45	3 h	Conventional heat	0.9
C-8	wheat straw	45	3 h	Conventional heat	1
C-9	barley straw	45	3 h	Conventional heat	0.8
C-10	rice hull	45	3 h	Conventional heat	0.7
CX-1	m-cellulose	45	30 min	microwave	0.7
CX-2	f-cellulose	45	30 min	microwave	0.8
CX-1a	m-cellulose	85	3 min	microwave	0.7
CX-2a	f-cellulose	85	3 min	microwave	1.1

**Table 3:** Typical reactions of cardanol with DEAD, all conducted at 70 °C with ethyl acetate solvent (adapted from ref. [98]).

Sample	Wt. Ratio: DEAD/Cardanol	Reaction Mode	Reaction Time
C-0	0	none	0
C-1	0.349	conventional heat	6 h
C-2	0.692	conventional heat	6 h
M-1	0.357	microwave	5 min
M-2	0.595	microwave	5 min

**Table 4:** Synthesis of polyurethane with different lactose/TDI stoichiometry and heating mode (conventional or microwave). All reactions were done with 1 g lactose and 5.5 mL DMF at 145 °C for 20 min (adapted from ref. [127]).

Sample	Weight Ratio TDI:Lactose	Heating Method	React Time	Product Yield %	Product Form in DMF
A	0.2	conventional	20 min	14	liquid
B	0.4	conventional	20 min	52	liquid
C	0.6	conventional	20 min	71	liquid
D	0.8	conventional	20 min	74	visc liquid
E	1	conventional	20 min	90	gel
F	1.2	conventional	20 min	96	hard gel
D′	0.8	microwave	3 min	76	visc liquid
E′	1	microwave	3 min	95	gel
F′	1.2	microwave	3 min	98	hard gel

**Table 5:** Comparison of total phenolics extracted (mg/g bean, GAE) from common beans through microwave extraction versus traditional extraction with water at 100 °C, Adapted from ref. [134].

Bean Type	Microwave Heat		Conventional Heat	
	Cotyledon	Hull	Cotyledon	Hull
Navy	5.48	3.45	1.71	1.76
Pinto	5.42	29.17	1.53	15.41
Small Red	5.77	39.98	1.74	20.35
Black	4.69	29.37	1.86	14.69
Great Northern	3.77	3.5	1.55	1.61
Pink	5.94	31.54	2.02	18.04
Light Red Kidney	5.44	24.54	2	12.48
Dark Red Kidney	4.66	25.62	2.03	12.41
*Average*	*5.15*	*23.40*	*1.81*	*12.09*

## Data Availability

No new data were created or analyzed in this study. Data sharing is not applicable to this article.
